# Exponentially Modified Peak Functions in Biomedical Sciences and Related Disciplines

**DOI:** 10.1155/2017/7925106

**Published:** 2017-06-05

**Authors:** A. Golubev

**Affiliations:** Saint-Petersburg State University, Saint Petersburg, Russia

## Abstract

In many cases relevant to biomedicine, a variable time, which features a certain distribution, is required for objects of interest to pass from an initial to an intermediate state, out of which they exit at random to a final state. In such cases, the distribution of variable times between exiting the initial and entering the final state must conform to the convolution of the first distribution and a negative exponential distribution. A common example is the exponentially modified Gaussian (EMG), which is widely used in chromatography for peak analysis and is long known as ex-Gaussian in psychophysiology, where it is applied to times from stimulus to response. In molecular and cell biology, EMG, compared with commonly used simple distributions, such as lognormal, gamma, and Wald, provides better fits to the variabilities of times between consecutive cell divisions and transcriptional bursts and has more straightforwardly interpreted parameters. However, since the range of definition of the Gaussian component of EMG is unlimited, data approximation with EMG may extend to the negative domain. This extension may seem negligible when the coefficient of variance of the Gaussian component is small but becomes considerable when the coefficient increases. Therefore, although in many cases an EMG may be an acceptable approximation of data, an exponentially modified nonnegative peak function, such as gamma-distribution, can make more sense in physical terms. In the present short review, EMG and exponentially modified gamma-distribution (EMGD) are discussed with regard to their applicability to data on cell cycle, gene expression, physiological responses to stimuli, and other cases, some of which may be interpreted as decision-making. In practical fitting terms, EMG and EMGD are equivalent in outperforming other functions; however, when the coefficient of variance of the Gaussian component of EMG is greater than ca. 0.4, EMGD is preferable.

## 1. Introduction

The normal (Gaussian) and the exponential are probably the most widely known distributions and quite ubiquitous, too. No wonder that situations are possible where they are expected to meet each other. The resulting composite distributions have been suggested to be relevant, for example, to times between consecutive cell divisions [1–4] in cell biology and to times from stimulus to response [5–8] in psychophysiology.

**Figure 1:**
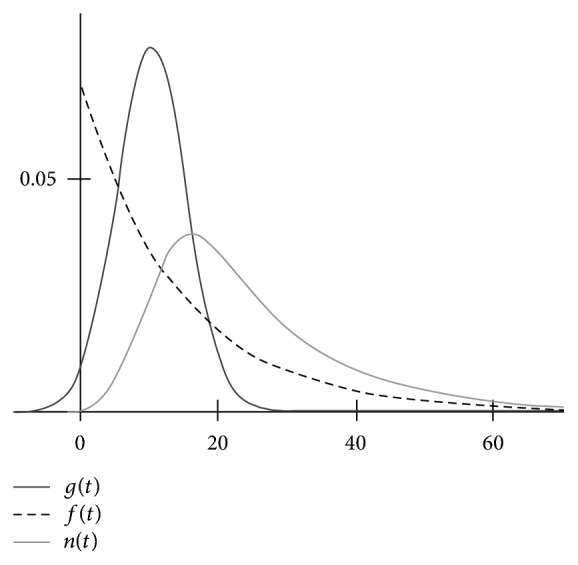
The plots of a Gaussian *g*(*t*) and an exponential distribution *f*(*t*) and of the result of their convolution *n*(*t*) (EMG) at *S* = 10, *σ* = 5, and *k* = 0.07. The plot may also be regarded as showing the result of the deconvolution of an EMG into its exponential *f*(*t*) and Gaussian *g*(*t*) components. The Gaussian component may significantly extend to the negative domain even if *n*(*t*) (EMG) may seem to be all nonnegative, which may be especially misleading if data points at small *t*, for example, within 5 time units, are lacking.

**Figure 2:**
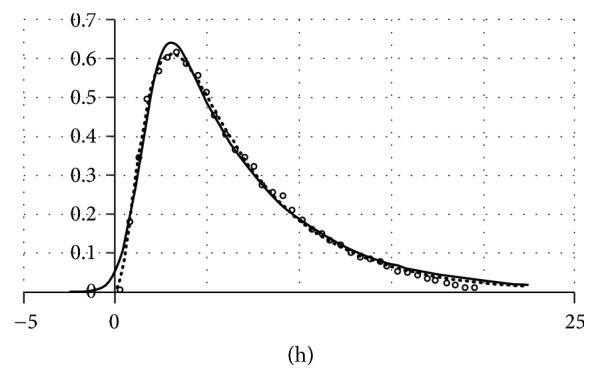
Data on intervals between the transcriptional bursts of the prolactin gene [19] approximated with EMG (solid line) and EMGD (dotted line). The data are shown with open circles. In the source paper [19], data were represented with histogram bars. The middle points of their tops are plotted here on an arbitrary vertical scale because only *X*-scale (time, h.) is what matters in the present context.

**Figure 3:**
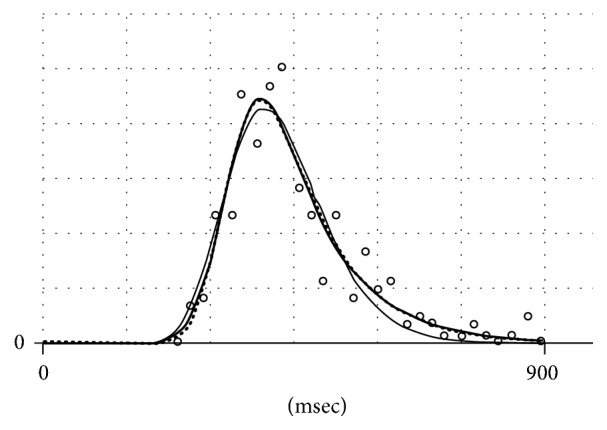
Data on response time distribution (RTD) picked up from [20] (open circles), which are approximated with EMG (thick solid line), EMGD (dots), and Wald distribution (thin solid line). The vertical scale is irrelevant.

Generally speaking, when the times of the passages of certain type objects from their initial to their final state are composed of variable times of their transition to an intermediate state and of their dwelling in the intermediate state, out of which they exit at random to their final state, then the random variable that represents the overall passage time of any such object is the sum of two independent random variables, the transit time and the dwell time, and the distribution of the overall passage times is defined as the convolution of the distributions of its summands [9]. The convolution of a Gaussian distribution and a negative exponential distribution is known as the exponentially modified Gaussian (EMG). Its generic plot is shown in Figure 1, and the approximations of empirical datasets with EMG are shown in Figures 2, 3, and 5.

EMG has been introduced about 40 years ago in chromatography (see [10]) and psychophysiology (see [5]). In both cases, each accounting for about 100 entries in the Scopus database, EMG seems preferable over other skewed distributions, such as lognormal, gamma, Weibull, and Wald (inverse Gaussian), not only because of its better formal fits to data but, also and no less importantly, because of its straightforwardly interpretable parameters.

More recently, EMG was suggested to be applicable to time distributions related to cell proliferation and differentiation [1, 2] and to distinct transcriptional states of active genes [2, 11]. Other novel EMG applications may be found in physiology [12], physics [13], and computer science [14].

However, there are cases when the deconvolution of an apparent EMG yields a Gaussian whose significant portion extends to the negative domain, which makes no physical sense. In such cases, nonnegative peak functions must be more appropriate for being convoluted with the negative exponent. In particular, a closed form expression for the exponentially modified gamma-distribution (EMGD) has been suggested and shown to be relevant to at least some of such cases [3]. In the present short review, the recent expansion of EMG applicability is considered in comparison with EMGD.

## 2. On Notations and Calculus

Exponentially modified functions result from applying the mathematical operator called convolution to nonmodified functions. The conventional definitions and notations for convolution are as follows:

(1) yxgx∗fx=∫−∞+∞fx′gx−x′dx′=∫−∞+∞fx−x′gx′dx′,

where *g*(*x*) is a nonmodified function; *f*(*x*) is, in the present context, a negative exponential function (however, generally speaking, it may be any function); *x*′ is an accessory (dummy) variable, which may be interpreted as the extent of shifting the plot of one function relative to the plot of the other function along the axis representing their common argument *x*; and *y*(*x*) captures the resulting changes in the common area under the two plots.

When the domain of any of the convoluted functions is other than (−*∞*, +*∞*), the limits of the convolution integral must be changed, respectively. Being treated in different mathematical contexts, for example, [15–17], these relationships may be a source of confusion, especially with regard to integration limits, in using EMG in biomedicine and related fields, for example, chromatography (see below Section 3.1).

In probability theory, convolution is used to define the probability density function (PDF) of the sum *h* of two independent stochastic variables, say *ξ*_1_ and *ξ*_2_. In this case, according to Feller [9, p. 6],

(2) $$\begin{array}{c} h\left(\;s\;\right)=\overset{+\infty }{\underset{-\infty }{\int }}f\left(\;y\;\right)g\left(\;s-y\;\right)dy, \end{array}$$

where *f* and *g* are the PDFs of *ξ*_1_ and *ξ*_2_, respectively, *y* is the value of the stochastic variable *ξ*_1_, and *s* is the value of the sum of the stochastic variables *ξ*_1_ + *ξ*_2_.

Integration limits in (2) are −*∞* and +*∞*. However, as pointed out, for example, in [9, p. 7] and [18, p. 216], integration limits must be different when at least one of the stochastic variables has nonzero probability densities only within a limited domain.

If probability densities of one of the variables are zero in the negative domain, then

(3) $$\begin{array}{c} h\left(\;s\;\right)=\overset{s}{\underset{-\infty }{\int }}f\left(\;y\;\right)g\left(\;s-y\;\right)dy. \end{array}$$

If probability densities of both variables are zero in the negative domain, then [9]

(4) $$\begin{array}{c} h\left(\;s\;\right)=\overset{s}{\underset{0}{\int }}f\left(\;y\;\right)g\left(\;s-y\;\right)dy. \end{array}$$

For the subsequent discussion, notations will be chosen to account for the biological meanings behind them. In particular, the stochastic variable is time *t* in most cases. Another point is that empirical distributions in biology are usually obtained starting from a counting procedure, for example, by counting of cells having intermitotic periods or of subjects showing response times within certain intervals of *t*. Therefore, the notation *n*(*t*) is chosen for the respective PDFs:

(5) $$\begin{array}{c} n\left(\;t\;\right)=\underset{\Delta t\to 0}{\lim}\left[\;\frac{N\left(t\right)-N\left(t-\Delta t\right)}{\Delta t}\;\right], \end{array}$$

where *N* is the normalized number of counts.

Using this notation, the convolution of a normal distribution and an exponential distribution is written as

(6) $$\begin{array}{c} n\left(\;t\;\right)=g\left(\;t\;\right)*f\left(\;t\;\right), \end{array}$$

where *t* is time (in the present context); *n*(*t*) is the probability density of events of interest; that is, *n*(*t*)d*t* is the normalized number of objects that have experienced this event in the time interval (*t*, *t* + d*t*), for example, the number of cells that, after having simultaneously emerged by the division of their mother cells, have divided within the time interval (*t*, *t* + d*t*); $g(t)=\left(1/\sigma \sqrt{2\pi }\right)\times \exp\;\left(-{\left(S-t\right)}^{2}/2\sigma^{2}\right)$ is Gaussian probability density function, its domain being (−*∞*, +*∞*), *S* being the mean of a Gaussian, and *σ*^2^ being the dispersion of a Gaussian; and *f*(*t*) = *k*exp⁡(−*kt*) is an exponential probability density function, its domain being (0, +*∞*) and *k* being the rate parameter (1/*k* is the mean of an exponential distribution and 1/*k*^2^ is its dispersion).

The computation of a closed form solution of *g*(*t*)*∗f*(*t*) using a dummy variable *t*′ (to make one of the convoluted functions shifted relative to the other by *t*′) starts with the integral:

(7) nt=gt∗ft=kσ2π∫−∞texp⁡−t−S−t′22σ2exp⁡−kt′dt′.

It may be solved by introducing the error function erf, which is not representable with elementary functions:

(8) $$\begin{array}{c} \mathrm{erf}\left(\;z\;\right)=\frac{2}{\sqrt{\pi }}\overset{z}{\underset{0}{\int }}e^{-t^{2}}dt. \end{array}$$

The resulting closed form solution of (7) is

(9) nt=kπ·exp⁡kS−t+kσ221−erf⁡S−t+kσ22σ.

A generic plot of EMG built according to (9) is shown in Figure 1, where the convoluted distributions are also shown. Notably, approximating an experimental dataset with (10) allows making estimates of the parameters of the convoluted functions, including their mean values, for example, mean transit time and mean dwell time (see Section 1).

The equivalents of (9) having multipliers to account for differences in peak height are inbuilt in curve-fitting software designed for chromatographic analysis, such as Fityk or SAS Fit, or for more general purposes, such as TableCuve2D, which is used throughout this paper.

A problem with (9) is that, strictly speaking, it is inapplicable to cases where negative time domains have no physical sense (in other words, above-zero *n*(*t*) at *t* = 0 is physically impossible). This relates to almost any biologically relevant situation where measured *t* values are times between sequential events, for example, from stimulus to response (Section 3.2), from cell birth to cell division (Section 3.3), or from termination to resumption of transcription at a defined active gene (Section 3.4).  Figure 2 illustrates this point using times from termination to resumption of transcription (see Section 3.4) as an example.

It has been suggested [3] that in such cases the gamma-distribution may be more appropriate for convolution with a negative exponential distribution. The result of the convolution may be termed as the exponentially modified gamma-distribution (EMGD).

The convolution integral for EMGD may be written as

(10) ntgt∗ft=∫0tft−t′·gt′·dt′=∫0te−t−t′/ττ·t′e−t′/bc−1bcΓc·dt′,

where *g*(*t*) = *t*^*c*−1^*e*^−*t*/*b*^/*b*^*c*^Γ(*c*) is the gamma-distribution, whose domain is (0, +*∞*), Γ(*c*) = ∫_0_^*∞*^*t*^*c*−1^*e*^−*t*^d*t* is the gamma-function, and *f*(*t*) is the negative exponential function.

The gamma-distribution is a generalization of the Erlang distribution *g*(*t*) = *t*^*c*−1^*e*^−*t*/*b*^/*b*^*c*^(*c* − 1)!, which is essentially the convolution of *c* identical exponential distributions whose 1/*k* = *b*. In the gamma-distribution, the values of *c* are not limited to integers.

The negative exponential component of (10) is expressed as *f*(*t*) = (1/*τ*)exp⁡(−(1/*τ*)*t*), where the dimensionality of *τ* is the same as that of *b*. Both, *τ* and *b*, are interpreted here as the mean (characteristic) times of objects dwelling in states out of which they exit at random. EMGD can approximate this situation when *τ* ≫ *b*_max_ ≫ (*b*_max_ − *b*_min_). The rationale for this condition is discussed in [3].

The solution of the improper integral by (10) may be written as

(11) nt=e−t/τ·b−c·τ−bτ·b−c·τ·Γc−1·Γc−Γc,τ−bτ·bt,

where Γ(*c*, ((*τ* − *b*)/(*τ* · *b*))*t*) = ∫_((*τ* − *b*)/(*τ* · *b*))·*t*_^*∞*^*e*^−*t*^ · *t*^*c*−1^ · d*t* is an upper (complementary) incomplete gamma-function.

Algorithms for numerical treatment of incomplete gamma-functions are included in standard curve-fitting software tools, such as TableCurve 2D used in the present paper and earlier [1–3].

Figure 2 illustrates the application of EMG and EMGD to fitting data on the duration of intervals between the transcriptional bursts of the prolactin gene.

Although fits to data according to the determination coefficient (*r*^2^) are very good and virtually identical for EMGD (0.997) and EMG (0.993), EMG extends to the negative domain and if treated as a PDF, it will not be integrated to unity on the physically valid domain (0, +*∞*); therefore, EMGD is preferable for physical reasons even though there are cases when its fit to transcriptional data is poorer than that of EMG [3].

With all that, data points related to an empirical distribution may be, and often are, located on the time axis so far from its origin that EMG and EMGD become equivalent as analytical approximations. This is illustrated in Figure 3 where data on distribution of times from stimulus to response in humans are shown (see Section 3.2 for more details).

**Figure 4:**
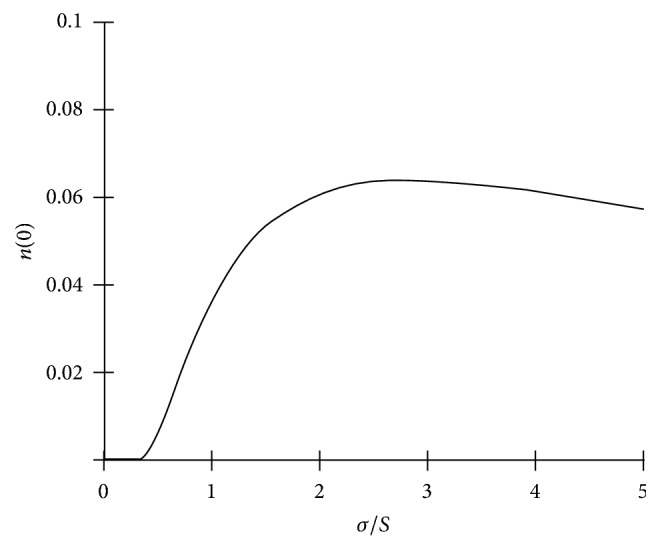
Increasing the height of the intersection of EMG (see (9)) with *y*-axis (*n* at *t* = 0) upon increasing the coefficient of variance (*σ*/*S*) of its Gaussian component. A greater height is associated with a more prominent extension of EMG to the negative domain.

In Figure 3, EMG and EMGD look virtually identical. This is no surprise because the distance of EMGD peak from the origin is defined by the parameter *c* of the gamma-distribution. With increasing *c*, the shape of a gamma-distribution approaches that of a Gaussian (in conformance with the central limit theorem). However, with decreasing the distance, no part of EMGD will extend to the negative domain; and *n*(*t*) at *t* = 0 will be zero. By contrast, with EMG, the interception of its plot with abscissa will increase with increasing the coefficient of variance of its Gaussian component, that is, the ratio of *σ* to *S*. The height of the interception of EMG plot with the ordinate will become appreciable at *σ*/*S* above 0.4 (Figure 4).

## 3. Applications and Implications of Exponentially Modified Peak Functions in Different Disciplines

### 3.1. Chromatography

In chromatography, EMG is employed since mid-1960s to describe chromatographic peaks, whose shape is presumably determined by the diffusion-caused Gaussian blur of a compound during its passage through a column and by extracolumn effects of its exponential dilution in a detector cell [10, 21, 22]. The superposition of these two processes will skew an almost Gaussian symmetrical peak, whose width depends on diffusion, into a peak, whose skewness depends on detector cell volume. Importantly, conformance to EMG was demonstrated for the absorbance profiles of the bands of substances during their passage through a column. In this case, extracolumn effects are excluded, and EMG may be thought of as being generated by a combination of processes that include substance transfer (transit time) by the liquid phase and substance retention (dwell time) by the solid phase, from which the substance dissociates by a first-order process [23].

Different authors suggest a bewildering variety of mathematical expressions for EMG to be used in chromatography. The list compiled by Di Marco and Bombi [10] includes 18 very differently looking functions, among which 12 are judged as essentially correct and equivalent to each other, except for representing different steps towards the closed form solution of the convolution integral of EMG and/or implying different integration limits (see Section 2).

Hanggi and Carr [21] discussed potentially important problems arising upon different assumptions concerning the integration limits and suggested a solution for EMG where the lower limit is assumed to be zero and the upper limit to be the independent variable. The rationale of this assumption is that the domain of any component function of a convolution used in chromatography must be nonnegative, otherwise meaningless above-zero concentration values at elution time *t* = 0 may be yielded when integration limits (−*∞*, *t*) are used to compute EMG, and these values may become practically considerable at *σ*/*S* greater than about 0.4 (see Figure 4). However, both approaches to integration limits are equivalent, in practice, at low *σ*/*S*.

At present, (9) modified by a multiplier accounting for peak amplitude is used in commercial software packages, such as TableCurve2D, PeakFit, Fityk, and SASfit, devised for curve fitting or peak analysis.

### 3.2. Psychophysiology

The first attempts to use EMG in a biological context, which date back to early 1960s, relate to response time distributions (RTD) in psychophysiological studies (reviewed in [6, 20]). However, probably because the result of convolution of a Gaussian and a negative exponential function has been termed ex-Gaussian in this field, this approach has been developed independently from EMG usage in chromatography, which has started at about the same time. The idea that the positively skewed distributions of times from stimulus to response are consistent with the ex-Gaussian is based on the premise that times-to-response may include exponentially distributed periods needed for making a decision to respond and normally distributed periods needed to respond according to the decision (the alternative possibility, that is, that decision time is distributed normally and execution time is distributed exponentially, is also considered [5]). The belief that ex-Gaussian (=EMG) is appropriate to RTD is evidenced by the development of dedicated software tools intended for finding best-fit EMG parameters of experimental RTD and for comparing EMG with other functions common in this field [20]. Remarkably, there has been no whatever crosstalk between chromatography and psychophysiology in designing software tools intended for very similar purposes.

The conformance of EMG and EMGD to an exemplary RTD presented in [20] is shown in Figure 3. The figure also shows an approximation of data with Wald (inverse Gaussian) distribution, which is also suggested for RTD data.

The use of EMG in psychophysiology was motivated by the intent to attribute a physiological significance to observed changes in RTD shapes. In particular, the centroid (*S*) was associated with the decision-making phase, whereas the rate constant (*k*) was associated with either the phase of execution of a decision or the phase or information processing required for a decision. The use of EMG was disputed by the proponents of another positively skewed PDF, that is, Wald (inverse Gaussian) distribution, based on other underlying premises rather than on a better or poorer fit (in the particular case shown in Figure 4, the fit of the Wald distribution is obviously inferior to those of EMG and EMGD). This controversy was discussed by Matzke and Wagenmakers [6] who concluded that EMG anyway remains a useful descriptive tool.

Some recent examples of using the deconvolution of EMG (ex-Gaussian) for distinguishing the different phases of psychophysical processes include cognitive changes that occur upon normal brain aging and Alzheimer disease [7] and in anxiety patients [24] and cocaine abusers [8] and variabilities found in word pronunciation time [25], eye fixation time during reading [26], and visual response time [5].

### 3.3. Cell Biology

It has been shown [1] that cell number distributions over times between sequential cell divisions (interdivision period, IDT) usually conform to EMG better than to traditionally used positively skewed distributions, such as lognormal or gamma. Subsequently, EMGD was shown to be no less appropriate as a descriptive model and more reasonable in biological (physical) terms, both EMG and EMGD outcompeting other positively skewed distributions [3].

The incentive to try EMG for fitting IDT was prompted by the transition probability model of cell cycle [27]. The model implies that, in a cycling cell population, daughter cells after having been born by their mother cell divisions will follow the courses of their cell cycles until reaching at different almost normally distributed times a state out which they exit at random; therefore, cell dwelling times in this state will be distributed exponentially. In a cell population, the exits of cells from this state will constitute a first-order process. If so, the overall IDT distribution in a cell population must be generated by the convolution of a Gaussian and a negative exponential function.

The intermediate state, which is mapped to G1 and is exited by cells at random, may be associated with the restriction point (RP) of cell cycle ([1, 2, 28] and references therein) and has been suggested to provide a time for cell fate to be determined, that is, whether a cell will pass its RP to divide in its current state or will become committed, upon passing its RP, to change its state so as to make itself or its progeny different, up to the loss of the ability to divide, such as upon a terminal differentiation [1, 2].

EMGD has been shown to approximate IDT distribution as well as EMG does [3]. In technical terms, EMG may be more convenient for IDT decomposition into the probabilistic and the deterministic part of the cell cycle according to the transition probability model. However, EMGD is more appropriate in physically meaningful terms [3].

### 3.4. Molecular Biology

EMG has been suggested to be relevant to possible transcriptional mechanisms of the events that are associated with the restriction point of the cell cycle and can determine cell fate [2, 11].

The events are thought to result from discrete random changes in gene activity. The rationale for this suggestion is detailed elsewhere [2, 11, 29] based on published data about the direct observation of nascent mRNA association with cyclin D1 gene in single cells [30, 31] and on inferences from mathematical analysis of protein level fluctuations in single cells [19, 32, 33], in combination with some common-sense reasoning [29]. The available evidence reviewed in [3, 11, 33] supports the view that the activity of, at least, some genes consists of random bouts of their engagement in mRNA production, which alternate with periods of gene idleness occurring at gene-specific mean frequencies. All authors who address this issue agree that the engagement periods, whatever they are called, are distributed exponentially consistent with that they are terminated by the dissociation of transcription preinitiation complexes of transcription factors from gene promoters. The distribution of idleness periods was suggested [32, 33] to be generated by a process consisting of two first-order steps, therefore being consistent with the convolution of two exponential functions. Checking published distribution histograms for their fits to different functions shows that the EMG and EMGD provide better fits than the exponentially modified exponent (EME) or simple gamma-distribution does [3]. However, EMG almost invariably showed above-zero intercepts of plots with ordinates (see Figure 2), which is hard to interpret in physically meaningful terms.

The process of preparation of an idle gene to the next bout of its transcriptional engagement, which is switched on by the complete assembly of a proper preinitiation complex at gene promoter, is likely to involve more than two steps, and this will make several sequential first-order processes at a cell population level. Therefore, the overall time distribution must conform to the convolution of several exponents, which if the exponents are identical, is described with the Erlang distribution, a particular case of the gamma-distribution. When one of the processes stands out of all others in having a far lower rate constant, the overall distribution will be the convolution of an exponent, which is generated by this process, with the distribution collectively generated by all other processes (see Section 2 and [3]). When a gamma-distribution is used for an Erlang distribution in the convolution with an exponent, the parameter *c* of the gamma-function may prompt an estimate of the number of steps required to resume transcription after its interruption at an active gene [3].

The same must be true for the cell cycle where reaching an RP-associated state is likely to be brought about by a combination of numerous loosely correlated events, each constituting, at the cell population level, a process having a much higher rate compared with the process associated with cell passage through the RP.

In either case, EMG- or EMGD-based analysis of time distributions makes it possible to distinguish influences on the slowest process, such as cell passage through RP, from influences on the other constituents of the overall process. With regard to cell proliferation, the importance of such distinction follows from the possibility of the involvement of cell dwelling in the RP-associated state in cell differentiation [1]. This approach has also been proposed to distinguish between the modes of action of different anticancer drugs [4].

### 3.5. Other Cases of Applicability of Exponentially Modified Peak Functions

The conformance of a distribution to an exponentially modified peak function is an indication that the underlying processes collectively generate transit times, whose distribution is peaked, and exponentially distributed dwell times and that the mean dwell time is much longer than the mean characteristic time of any of the processes that determine the transit time. It has been shown [1] that EMG does not perform well when there are no reasons to expect that a distribution may be generated in that way. However, situations where such reasons do exist must be quite common. This ubiquity, which is not limited to the areas discussed above, is illustrated below with cases where fits to EMG and/or EMGD are observed outside the original fields of the applicability of these distributions.

In a study of microtubule growth in living cells by confocal fluorescence microscopy, the profiles of fluorescence intensity of complexes of microtubule-end binding protein with green fluorescent protein were fit to EMG based on the premise that these profiles are generated by a convolution of exponential decay with point Gaussian blur associated with microscopy [34]. Indeed, it may be confirmed that the profiles presented in [34] correspond to EMG better than to other positively skewed peak functions (not shown).

EMG was used to model human skin conductance changes in response to stimuli, such as noise or image [12]. Both EMG and EMGD may be found at the top of ranked lists of fits of different peak functions to skin conductance versus time plots (not shown).

**Figure 5:**
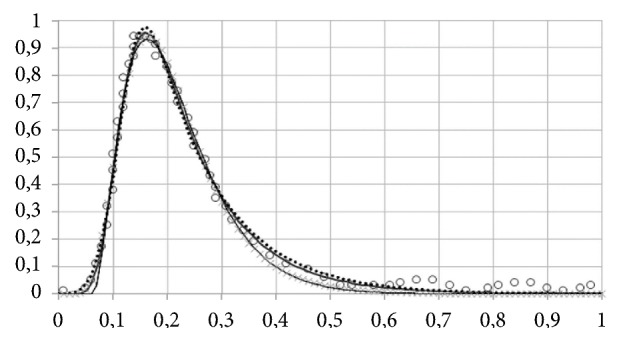
Fitting data on an amperometric spike produced by adrenaline release from adrenal medulla cell synaptic vesicle. Data points (open circles) are obtained by manual tracking of an amperometric trace presented in [36]. Scales are arbitrary. EMGD: thick solid line; EMG: dotted line; shifted gamma-distribution: thin solid line; lognormal distribution: symbols ×. Fits by determination coefficient (*r*^2^): EMGD (0.993) > EMG (0.991) > lognormal = shifted gamma (0.990).

In several studies of neurosecretion [35, 36], it was assumed that amperometric spikes, which result from the release of secreted substances from exocytotic vesicles, can be described by the convolution of a Gaussian with a decreasing exponential, which is partially governed by the diffusion of the substances towards the electrode.  Figure 5 shows that such spikes may be fitted with EMG and EMGD equally well.

More remote to the initial field of EMG applicability are studies where EMG was used to model channel holding time distribution in public telephony systems [37] and packet payload lengths for two-player first-person shooter games in the server-to-client direction [14]. In physics, EMG emerged in an analysis of excited state lifetime distributions [13] and was used to describe ion flight time distribution in a detector [38].

Searching for other cases where EMG may be applicable to time distributions for reasons discussed above shows that this may be true for some situation related to city traffic, such as that described in the paper [39] where the distribution of bus dwelling time at bus stops was fitted with a lognormal function; however, a better fit according to several goodness-of-fit criteria is obtained with EMG and EMGD (not shown).

## 4. Discussion

Making inferences from the shapes of distributions of variable parameters is a routine approach in physics; however, it has long been limited in biomedicine by enormous amounts of calculations. The state of things changes in recent years due to the availability of user-friendly software designed for this purpose.

A significant, in this regard, difference between biology and physics is that, in physics, distributions are often observed directly, whereas in biology they are generated by counting procedures, such as single-cell tracking, which may be very time-consuming and laborious. This problem will hopefully be ameliorated with the advent of novel automated single-cell tracking and other single-event monitoring techniques, which are increasingly introduced in biomedicine.

In the biological context, EMG and EMGD are useful as models, which suggest that, in a population of some objects there is, among the processes that generate the distribution of times required for each of the objects to pass between two observed consecutive states, a first-order process whose rate constant is much lower compared with those of all other processes. This slow process may be regarded as being generated by random discrete events that occur at a frequency making the mean interevent time comparable with the mean time between the two apparent consecutive states of the objects in question [1–3]. This interevent time is consistent with object dwelling in an unobservable intermediate state between the two observed states. Both, EMG and EMGD, make it possible to estimate the rate constant of this slow process, that is, the mean time of object dwelling in the intermediate state, and the contribution of all other processes to the overall time between the initial and final states and to its variance. Other similarly looking skewed distributions, such as lognormal or gamma, are not adequate to such cases.

However, no model can account for everything; therefore, any model is a compromise between physical (biological) relevance and mathematical tractability. To reiterate the epigraph to this writing: “… all models are wrong; the practical question is how wrong do they have to be to not be useful” (G. E. P. Box and N. R. Draper, 1987, p. 74). In this regard, EMG, which extends to the negative domain, is essentially wrong when it is applied to interevent time distributions; however, because of its easier mathematical tractability and the availability of ready-to-use curve-fitting tools, it is still useful as a descriptive option when the variation coefficient of its Gaussian component is low, within ca. 0.4. When the coefficient is higher than that, the use of EMGD is warranted. The failure of a distribution related to a class of phenomena, to which EMG or EMGD has been found to be generally applicable, to conform to them in a specific case may prompt that there is either a methodological bias or some unrecognized factor at work.

The use of EMG and/or EMGD in cell biology may help to supplement the assessment of the duration of different phases of cell cycle (G1, S, and G2) based on DNA content distributions with the assessment of the probabilistic and the deterministic part of cell cycle, according to the transition probability model, based on interdivision time distributions [1, 2]. This approach was used to discriminate between the modes of action of anticancer drugs [3, 4]. The importance of such discrimination follows from the suggestion that the rate of cell differentiation depends on the mean probabilistic rather than deterministic part duration, the former being associated with the restriction point of the cell cycle and suggested to provide cells with time for making a decision, by random choice, whether to continue proliferation or to become committed to withdrawal from the cell cycle [1].

Similarly, in psychophysiology, EMG or EMGD may help to distinguish between a phase required to make a decision to respond to a stimulus and a phase required to either execute the decision or to become prepared to make it [5, 6].

Based on the apparent pervasiveness of exponentially modified peak functions, such as EMG and EMGD, in different biological context—from molecular through cellular to physiological—it is tempting to suggest that this is a reflection of a common biological strategy of making decisions, for example, whether to commence or not to a next burst of gene transcription, cycle of cell proliferation, or response to a stimulus, each time there being alternative options chosen at random. As far as the probabilities of such choices depend on conditions, a population of biological systems can thus come to an optimal balance of different ways of coping with the unforeseeably changing world around them.
